# mPEG-functionalized polyadipate triblock copolymers as promising nanocarriers for the controlled delivery of therapeutic agents in breast cancer treatment

**DOI:** 10.1007/s00210-026-05054-w

**Published:** 2026-02-10

**Authors:** Nazan Gökşen Tosun, Seçil Erden Tayhan, İsa Gökçe, Cemil Alkan

**Affiliations:** 1 Department of Medical Services and Techniques, Tokat Vocational School of Health Sciences, Tokat Gaziosmanpaşa University, Tokat, Turkey; 2 Department of Pharmaceutical Biotechnology, Pharmacy Faculty, Tokat Gaziosmanpaşa University, Tokat, Turkey; 3 Department of Biochemistry, Pharmacy Faculty, Tokat Gaziosmanpaşa University, Tokat, Turkey; 4 Department of Chemistry, Science and Letters Faculty, Tokat Gaziosmanpaşa University, Tokat, Turkey

**Keywords:** Nanocarriers, Polyadipate, PEGylation, Triblock copolymer, Doxorubicin, Breast cancer

## Abstract

The increasing incidence of breast cancer is leading researchers to investigate new treatment approaches. Targeted therapy approaches are particularly attractive because they minimize the detrimental effects of therapeutic agents on healthy tissues and cells by focusing on tumor sites. This study focuses on synthesizing mPEG-modified triblock copolymers as carrier materials for drug delivery applications, enabling the efficient encapsulation of DOX, and evaluating the cytotoxic effects of the resulting nanocarriers on breast cancer cell lines. In this study, mPEG-poly(butylene adipate)-mPEG and mPEG-poly(ethylene adipate)-mPEG triblock copolymers were synthesized by a step-growth polycondensation polymerization method. Firstly, poly(butylene adipate) (pBAd) and poly(ethylene adipate) (pEAd) were synthesized to form the body of the triblock copolymer, and their chemical structures were characterized using Fourier transform infrared (FT-IR) and ^1^H NMR spectroscopy. The end-group analysis method was applied to determine the average molecular weights of the pBAd and pEAd polymers before their modification with mPEG-500. The nanocarriers produced by the double emulsion method were analyzed using the dynamic light scattering (DLS) method, while encapsulation efficiency and the DOX release profile were measured using a spectrofluorometer. The antiproliferative effects and cellular uptake capacities of the resulting nanocarriers were subsequently examined in MCF-7 and MDA-MB-231 cells. The cytotoxicity of DBANP and DEANP nanocarriers was lower than that of free DOX, demonstrating that encapsulation reduces drug-associated toxicity and may enhance safety. These findings suggest that the nanocarrier systems developed in this study show strong potential as promising candidates for breast cancer therapy.

## Introduction

Breast cancer, despite being one of the most extensively studied types of cancer, continues to be a strong contender among global health issues due to its complex pathogenesis (Xiong et al. 2025; Kim et al. 2025). Traditional methods such as surgical resection, chemotherapy, and radiotherapy are limited in their applicability due to their severe side effects on healthy cells, their weakening effect on the immune system, their allergenic effects, the development of high drug resistance, and the aesthetic damage that further weakens the patient psychologically. The unsustainability of traditional methods necessitates ongoing research to treat and prevent breast cancer (Gökşen Tosun and Kaplan 2025; Spriet et al. 2025). In this context, especially targeted therapies are providing solutions to the shortcomings of conventional treatment. Drug delivery systems, particularly those engineered at the nanoscale, have garnered the interest of researchers due to their ability to target cancer cells while minimizing the toxicity caused by chemotherapeutic agents in healthy cells and tissues (Saripilli and Sharma 2025; Li et al. 2025). Drug delivery systems, which are either nano- or microscale, continue to utilize natural polymers, synthetic polymers, and both organic and inorganic materials in encapsulating drugs. The two most important criteria for selecting materials for drug delivery systems are their biocompatibility and biodegradability (Musielak and Krajka-Kuźniak 2025; Beach et al. 2024; Guo et al. 2025a, 2025b; Sabzehali 2025; Panda et al. 2024).

Polymeric nanocarriers are emerging as versatile nanoplatforms among drug delivery systems due to their biocompatibility and biodegradability, as well as their ability to enhance drug solubility, protect unstable therapeutic agents, and provide controlled release profiles. Among these systems, amphiphilic block copolymers are particularly attractive because they can spontaneously assemble into nanoscale structures with distinct hydrophilic and hydrophobic domains, allowing for the encapsulation of a wide range of therapeutic molecules (Mai and Eisenberg 2012; Discher and Eisenberg 2002). Compared to conventional diblock copolymers, amphiphilic triblock copolymers offer additional structural advantages arising from their symmetric architecture. The presence of hydrophilic blocks at both chain ends enhances interfacial stabilization, promoting the formation of more stable nanostructures. These features are especially relevant for emulsion-based nanoparticle preparation methods, where polymer architecture plays a critical role in determining particle size, colloidal stability, and drug retention (Mai and Eisenberg 2012; Discher and Eisenberg 2002; Alexandridis and Lindman 2000; Kataoka et al. 2001).

Aliphatic polyesters such as polyadipates have attracted increasing attention as hydrophobic segments in polymeric nanocarriers due to their flexibility, favorable compatibility with hydrophobic drugs, and tunable chain length (Torchilin 2001; Bu et al. 2019; Chesterman et al. 2020; Banik et al. 2016). Moreover, their biocompatibility and biodegradability make them unique for use in delivery systems. However, the most significant limitation of adipates is their hydrophobic nature (Ward and Jones 2011; Axioti et al. 2025; Mi et al. 2025; Das et al. 2025). To overcome this limitation, polyesters are PEGylated to impart hydrophilic properties and to ensure prolonged circulation in the bloodstream. These amphiphilic polymers, produced through the PEGylation of polyesters, are preferred in biomedical and pharmaceutical applications due to their adjustable physicochemical properties (Long et al. 2025; Chime et al. 2025; Makharadze et al. 2025). Therefore, this study aimed to develop polyadipate-based drug delivery systems that combine biocompatibility, biodegradability, tumor-site accumulation, and prolonged circulation through the use of PEGylation. To elucidate the effect of polymer chain structure and flexibility, adipate derivatives were synthesized using two diols with distinct characteristics: ethylene glycol and 1,4-butanediol. By employing the same amphiphilic triblock copolymer strategy, the influence of polymer architecture on the physicochemical properties, drug encapsulation behavior, and release characteristics of the resulting nanocarriers was systematically investigated.

Within this scope, amphiphilic polyadipate-based triblock copolymers were utilized to fabricate polymeric nanoparticles via a double-emulsion solvent evaporation method. Doxorubicin was selected as a model chemotherapeutic agent to evaluate passive targeting and controlled release behavior. The prepared nanocarriers were characterized in terms of chemical structure, particle size, surface charge, drug encapsulation efficiency, and in vitro biological performance, including antiproliferative activity and cellular uptake. Overall, the results highlight the potential of polyadipate-based triblock copolymers as a promising platform for breast cancer drug delivery applications.

## Materials and methods

### Materials

Adipic acid (99 + %), 1,4 butanediol (98 + %), ethylene glycol poly(ethylene glycol) methyl ether (mPEG-550) (99 + %), dibutyltin dilaurate (DBL) (95 + %), N, N-dimethylacetamide (DMAC) (99 + %), dichloromethane (DCM) (99.5 + %), hexamethylene di-isocyanate (HMDI) (99 + %), and indocyanine green (ICG) (99 + %) were purchased from Merck KGaA, Darmstadt, Germany. PVA (poly(vinyl alcohol)) (MW 6 kDa), used as a surfactant in this work, was commercially available from Polysciences, Inc., 400 Valley Road, Warrington, PA, USA.

DOX HCl, used for encapsulation into delivery carriers as a form of chemotherapy drug for breast cancer treatment, was purchased from Gold Biotechnology Inc., USA. For these in vitro cell culture studies, heat-inactivated fetal bovine serum (FBS), RPMI-1640 medium, Dulbecco’s Modified Eagle’s medium (DMEM), gentamicin solution, L-glutamine, phosphate-buffered saline (PBS), and trypsin–EDTA were all purchased from Biological Industries, Kibbutz Beit-Haemek, Israel. 3-(4,5dimethylthiazol-2-yl)−2,5–2,5-diphenyltetrazolium bromide (MTT) was obtained from Serva, Germany. The MCF-7 and MDA-MB-231 cell lines, used in cell culture studies, were purchased from the American Type Culture Collection.

### Synthesis of pBAd and pEAd

The polyadipates produced in this study (pBAd and pEAd), which will form the backbone of amphiphilic tri-copolymers, were synthesized under 2–3-mmHg conditions in the absence of both a catalyst and a solvent (Kaplan et al. 2023; Alkan et al. 2023). The reaction was carried out in a conical, double-necked flask fitted with a PTFE valve and connected to a vacuum motor, operating under a pressure of 2–3 mmHg. First, adipic acid (5.00 g, 34.21 mmol) was liquefied in the flask under a pressure of 2–3 mmHg. Subsequently, 1,4-butanediol (3.12 g, 41.06 mmol) was added at a dicarboxylic acid: diol molar ratio of 1:1.2 to synthesize pBAd. The reaction was continued under a nitrogen atmosphere, with stirring using a magnetic stirrer for 2–3 h. After the polycondensation reaction was completed, the viscous polymer melt was allowed to cool to room temperature and then, as the first step of the purification process, the viscous polymer was dissolved in acetone to remove unreacted monomers and low molecular weight oligomeric species. In the second step, the precipitation step, the polymer solution was precipitated by the gradual addition of cold ethyl alcohol under continuous stirring, resulting in the formation of a solid polymer phase. This precipitation-wash step was repeated twice to ensure effective purification. Filtration was performed to collect the purified polymer, and it was then dried by lyophilization until a constant weight was achieved. To synthesize pEAd, ethylene glycol was added to the reaction medium after adipic acid had melted in the flask. The reaction conditions, process, purification, and lyophilization were applied in the same manner as those for pBAd synthesis.

### Synthesis of the amphiphilic triblock copolymers

The hydrophobic polyesters pBAd and pEAd, which constitute the core segment of the amphiphilic triblock copolymers, were conjugated to both termini of the hydrophilic mPEG-550 via PEGylation. In this process, mPEG-550 (0.017 g, 0.23 mmol) and HMDI (0.039 g, 0.23 mmol) were dissolved in DMAC at a 1:1 molar ratio under ambient conditions, using a magnetic stirrer in a sealed, light-protected glass reactor. Subsequently, DBL was introduced as a catalyst, and the reaction was allowed to proceed for 48 h. The resulting mPEG-550–HMDI intermediate was synthesized separately for each adipate type (pBAd and pEAd), based on their respective molecular weights. Briefly, hydroxyl-terminated pBAd (2.19 g, 2.30 mmol, calculated based on end-group analysis) or pEAd (2.00 g, 2.30 mmol, calculated based on end-group analysis) was prepared for the reaction mixture at a polyadipates: mPEG–HMDI molar ratio of 1:0.1. The mPEG-550–HMDI intermediate was then added dropwise to the polyadipates, and the reaction mixture was stirred at room temperature for 72 h.

### Development of polyadipate-based nanoparticles

Polyadipate-based nanoparticles (BANPs and EANPs) were fabricated via the double emulsion solvent evaporation method (Kaplan et al. 2024). DCM was used as the organic solvent, and PVA served as the stabilizing surfactant.

Initially, the amphiphilic triblock copolymer (mPEG-pBAd-mPEG or mPEG-pEAd-mPEG, 50 mg) was dissolved in DCM at a final concentration of 10 mg/mL. To form the primary water-in-oil (W/O) emulsion, an aqueous phase was added dropwise to the organic polymer solution under continuous stirring, followed by probe sonication at 100% amplitude for 9 cycles, totaling 60 s.

The secondary water-in-oil-in-water (W/O/W) emulsion was formed by slowly adding the primary emulsion to 10 mL of 1.2% (w/v) PVA solution under magnetic stirring. This mixture was then subjected to probe sonication at 100% amplitude for 9 cycles over a total of 3 min. Subsequently, the resulting double emulsion was diluted by dropwise addition to 40 mL of 0.1% (w/v) PVA solution. Solvent evaporation was carried out by stirring the emulsion at 800 rpm at room temperature until complete removal of DCM.

The first step in the purification process was centrifugation at 10,000 rpm for 8 min. The purpose of this step is to remove excess PVA and unencapsulated material from the nanoparticle suspension by washing it with distilled water and then freeze-drying (lyophilization) the resulting pure nanocarriers for storage.

### Development of DOX-loaded polyadipate-based nanoparticles

To prepare DOX-loaded polyadipate-based nanoparticles (DBANPs and DEANPs), amphiphilic triblock copolymers (mPEG-pBAd-mPEG or mPEG-pEAd-mPEG, 50 mg) were first dissolved in DCM to achieve a final concentration of 10 mg/mL. Separately, a solution of the chemotherapeutic agent doxorubicin (DOX) was prepared in a glass flask at a final concentration of 10 mg/mL. From this solution, 50 µL was added to the copolymer solution. The mixture was stirred at 2020 rpm for 60 min to allow for encapsulation of DOX into the copolymer matrix.

Following encapsulation, 1 mL of distilled water was added dropwise to the organic phase to form the primary water-in-oil (W/O) emulsion, which was then subjected to probe sonication. To generate the secondary water-in-oil-in-water (W/O/W) emulsion, the primary emulsion was added dropwise to 10 mL of a 1.2% (w/v) PVA solution and was again sonicated under the same conditions.

Finally, the resulting DOX-loaded nanoparticles were collected by centrifugation, and the residual solvent was removed. The nanoparticle pellet was washed three times with distilled water to eliminate unencapsulated DOX and excess surfactant and then freeze-dried for storage.

### Chemical and physical characterization of polyadipates and amphiphilic triblock copolymers

The successful chemical synthesis of the polyadipates (pBAd and pEAd) and the amphiphilic triblock copolymers (mPEG-pBAd-mPEG and mPEG-pEAd-mPEG) was confirmed by FT-IR spectroscopy. FT-IR spectra of the synthesized polymers were recorded using a Jasco FT-IR 4700 spectrometer equipped with a compatible attenuated total reflectance (ATR) accessory. All measurements were performed in the wavenumber range of 400–4000 cm⁻^1^ at room temperature. For each sample, spectra were collected by averaging 16 scans to improve the signal-to-noise ratio. The use of the ATR accessory enabled the direct analysis of solid polymer samples without any additional sample preparation. The obtained spectra were employed to confirm the chemical structure of the polymers and to monitor functional group transformations occurring during copolymer synthesis.

^1^H NMR spectroscopy was employed to confirm the chemical structure of the synthesized amphiphilic triblock copolymers. The 1H NMR spectra of mPEG-pBAd-mPEG and mPEG-pEAd-mPEG were recorded at 400 MHz using a Bruker NMR spectrometer at room temperature, with deuterated chloroform (CDCl₃) as the solvent. Chemical shifts (δ) are reported in parts per million (ppm) relative to tetramethylsilane (TMS) as the internal standard. The characteristic proton resonances corresponding to the polyadipate backbone, diol segments, and PEG blocks were assigned based on their chemical environments to verify successful triblock copolymer formation.

The average molecular weights of the produced polyadipates and amphiphilic triblock copolymers were calculated using end-group analysis (Chaudhary et al. 1997). Briefly, the number of molecular weights of pBAd and pEAd polymers with hydroxyl end groups was determined using a titrimetric method based on end-group conversion. The intensity of the − OH groups’ peaks of terminal polymeric groups is considerably lower because the synthesis converts most of the − OH groups to esters. For this purpose, the –OH groups at the ends of the polymer chains were converted into carboxylic acid functional groups by reacting them with pyromellitic dianhydride (PMDA) under controlled conditions. One of the anhydride rings of PMDA was covalently bonded to the chain end by undergoing an esterification reaction with the hydroxyl end group of the polymer; the small amount of water present in the reaction medium under controlled conditions caused the hydrolysis of the second anhydride functional group of PMDA, leading to the formation of additional carboxylic acid groups. Thus, the pBAd and pEAd chain ends, which initially had hydroxyl functional groups, were converted into carboxylic acid end groups suitable for titration. The amount of the resulting acid end groups was quantitatively determined by acid–base titration with a standard base solution, and the number-average molecular weights (*M*ₙ) of the polymers were calculated using the measured acid values. This method eliminates uncertainties in the direct determination of hydroxyl end groups, allowing for the reliable determination of the number average molecular weights of pBAd and pEAd polyols.

### Characterization of polyadipate-based nanoparticles

The physicochemical properties of the non-loaded polyadipate-based nanoparticles (BANPs and EANPs), DOX-loaded nanoparticles (DBANPs and DEANPs), and ICG-loaded nanoparticles (IBANPs and IEANPs), including particle size, polydispersity index (PDI), and zeta potential, were determined using DLS.

The encapsulation efficiency (EE%) of DOX within the amphiphilic triblock copolymers (mPEG-pBAd-mPEG and mPEG-pEAd-mPEG) was quantified using a spectrofluorometer. The percentage of encapsulated DOX was calculated using the encapsulation efficiency formula reported in the literature. After centrifugation, fluorescence measurements were performed using a spectrofluorometer to determine the amount of unencapsulated DOX in the supernatant solution.

Encapsulation efficiency was calculated according to the following equation, as reported in the literature (Ghezzi et al. 2021).

$$\text{EE (\%)}=\frac{{W}_{\text{total DOX}}-{W}_{\text{free DOX}}}{{W}_{\text{total DOX}}}\times 100$$

where ${W}_{\text{total DOX}}$ is the total amount of DOX initially added and ${W}_{\text{free DOX}}$ is the amount of DOX detected in the supernatant.

Drug loading (DL%) values were calculated based on the amount of encapsulated DOX relative to the total nanoparticle mass, using the same experimental dataset employed for determining encapsulation efficiency. DL% values were also calculated according to the following equation, as reported in the literature (Yuan et al. 2018).

Drug loading (DL%) was calculated using the following equation:

$$\text{DL (\%)}=\frac{{W}_{\text{encapsulated DOX}}}{{W}_{\mathrm{nanoparticles}}}\times 100$$

where ${W}_{\text{encapsulated DOX}}$ represents the amount of DOX encapsulated within the nanocarriers and ${W}_{\mathrm{nanoparticles}}$ is the total mass of the recovered nanoparticles.

The release behaviors of DOX-loaded polyadipate-based nanoparticles (DBANPs and DEANPs) were evaluated by a spectrofluorometer. DBANPs and DEANPs were suspended in serum to simulate physiological conditions after production. After the samples were incubated for certain time intervals, the supernatant was collected and analyzed to monitor DOX release. The fluorescence intensity of the supernatant was measured, and the release profiles were recorded according to the absorbance of DOX released over time.

### In vitro cellular evaluation of polyadipate-based nanoparticles

#### Cell culture

In the current study, two different breast cancer cell lines were used to evaluate biological activity. One of them, the MCF-7 (ER +) cell line, was cultured in RPMI-1640 medium supplemented with 10% FBS, while the other, MDA-MB-231 (TNBC), was maintained in DMEM high glucose containing 10% FBS. Incubation was performed at 37 °C in a humidified atmosphere containing 5% CO_2_ for all cultures (Gökşen Tosun 2024).

#### In vitro antiproliferative evaluation of polyadipate-based nanoparticles

The antiproliferative activities of non-loaded polyadipate-based nanoparticles (BANPs and EANPs), DOX-loaded nanoparticles (DBANPs and DEANPs), and ICG-loaded nanoparticles (IBANPs and IEANPs) in breast cancer cell lines MCF-7 and MDA-MB-231 were determined by the MTT assay (Erden Tayhan 2024). Here, DOX was used as the chemotherapeutic agent, while indocyanine green (ICG) was preferred as a fluorescent dye to provide evidence in cellular uptake assays.

Initially, MCF-7 and MDA-MB-231 cells were seeded into 96-well plates at a density of 5 × 10^4^ cells/mL and incubated under standard culture conditions (37 °C, 5% CO_2_) for 24 h to allow for attachment. Since the cytotoxicity profiles of free DOX and ICG were previously evaluated (Gökşen Tosun et al. 2023), only the nanoparticle-encapsulated forms were tested in the current study. Following incubation, the cells were treated with BANPs, EANPs, DBANPs, DEANPs, IBANPs, or IEANPs at concentrations ranging from 1.25 to 40 µg/mL and then incubated for an additional 72 h. At the 24-, 48-, and 72-h time points, MTT solution (5 mg/mL in PBS) was added to each well and incubated at 37 °C for 3 h. After the resulting formazan crystals, formed by metabolically active cells, were dissolved in DMSO, the absorbance was measured at 570 nm using a microplate reader. Data analysis and graph plotting were accomplished using GraphPad Prism 8.0 software. All experiments were conducted in triplicate, and results are presented as mean ± standard deviation (SD).

#### In vitro cellular uptake of polyadipate-based nanoparticles

Cellular uptake of polyadipate-based nanoparticles in the MCF-7 and MDA-MB-231 breast cancer cell lines was determined using the chemotherapeutic agent DOX and ICG as a fluorescent dye. In vitro cellular uptake of ICG-loaded polyadipate-based nanoparticles (IBANPs and IEANPs) was visualized using inverted fluorescence microscopy, which utilizes the intrinsic fluorescence emission of ICG to assess the cellular uptake efficiency.

Before performing the cellular uptake assay, the antiproliferative activity of IBANPs and IEANPs was evaluated by the MTT assay. Cellular uptake studies were conducted based on the IC_50_ concentrations determined by the MTT assay. MCF-7 and MDA-MB-231 cells were seeded in 12-well plates at a density of 5 × 10^4^ cells/mL in each well and incubated under standard culture conditions (37 °C, 5% CO_2_) for 24 h. After incubation, the culture medium was removed, and the cells were treated with IBANPs, IEANPs, and free-ICG, each prepared in 1 × DPBS at its respective IC_50_ concentration. The cells were then incubated under the same conditions for an additional 4 h. Following incubation, the wells were gently washed with DPBS to remove unincorporated nanoparticles or dye. Cellular uptake was then examined using an inverted fluorescence microscope (Olympus CKX53), and images were recorded.

### Statistical analysis

All experimental data in the presented cell culture assay were statistically analyzed using GraphPad Prism 8.0 software. Two-way analysis of variance (ANOVA) was used to compare multiple groups, followed by post hoc Dunnett’s and Sidak’s multiple comparison tests where appropriate. Results with a *p* value of less than 0.05 (*p* < 0.05) were considered statistically significant.

## Results and discussion

### Characterization of the polyadipates and amphiphilic triblock copolymers

**Fig. 1:**
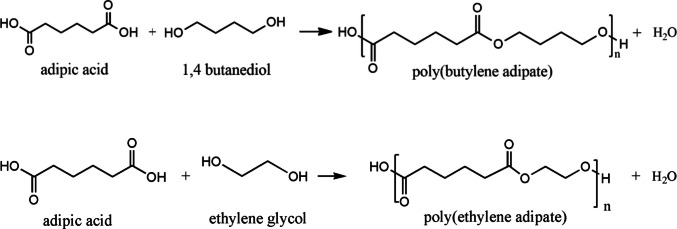
Chemical reaction scheme of the synthesis of pBAd and pEAd polyadipates

The hydrophobic parts of the amphiphilic triblock copolymers were synthesized by esterification, and the synthesis scheme of pBAd and pEAd was drawn, as shown in Fig. 1.

**Fig. 2:**
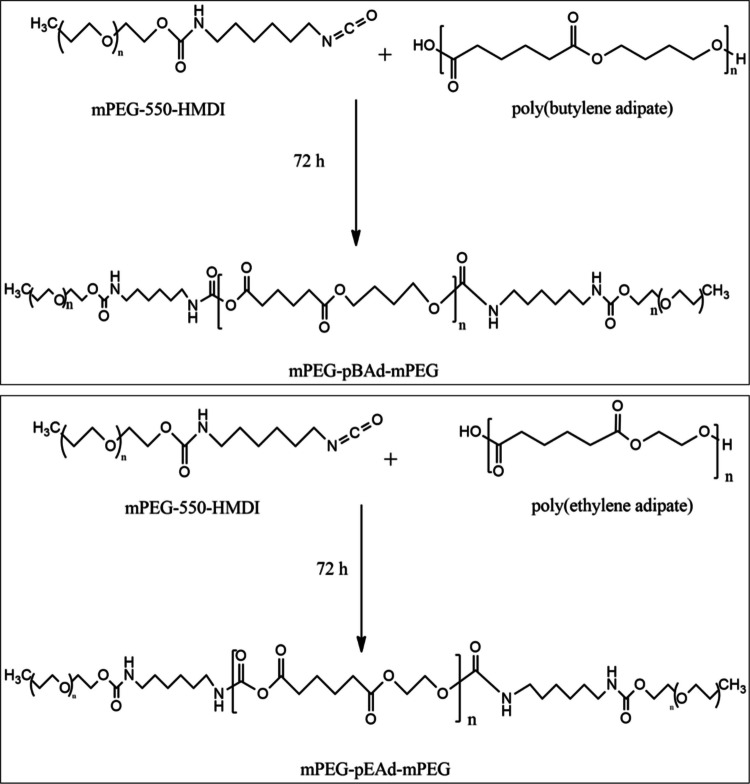
The schemes for mPEG-functionalization of pBAd and pEAd polyadipates

After the synthesis of the pBAd and pEAd esters, the determination of the number-average molecular weights of these esters enabled the calculation of their number-average molecular weights by the end-group analysis method due to the –OH termination of the end groups of the polymer chains. Therefore, the number-average molecular weights of the pBAd and pEAd esters were calculated as 952 g/mol and 870 g/mol, respectively. Having determined the average molecular weights of the esters, we PEGylated the pBAd and pEAd via conjugation with mPEG-550 through diisocyanate linkers. The PEGylation reaction of the polyadipates is illustrated schematically in Fig. 2. The average molecular weights of the triblock copolymers were calculated by including the molecular contributions of the mPEG blocks at both chain ends. The resulting values were rounded to the nearest higher average, yielding approximate molecular weights of 1700 and 2000 g/mol.

**Fig. 3:**
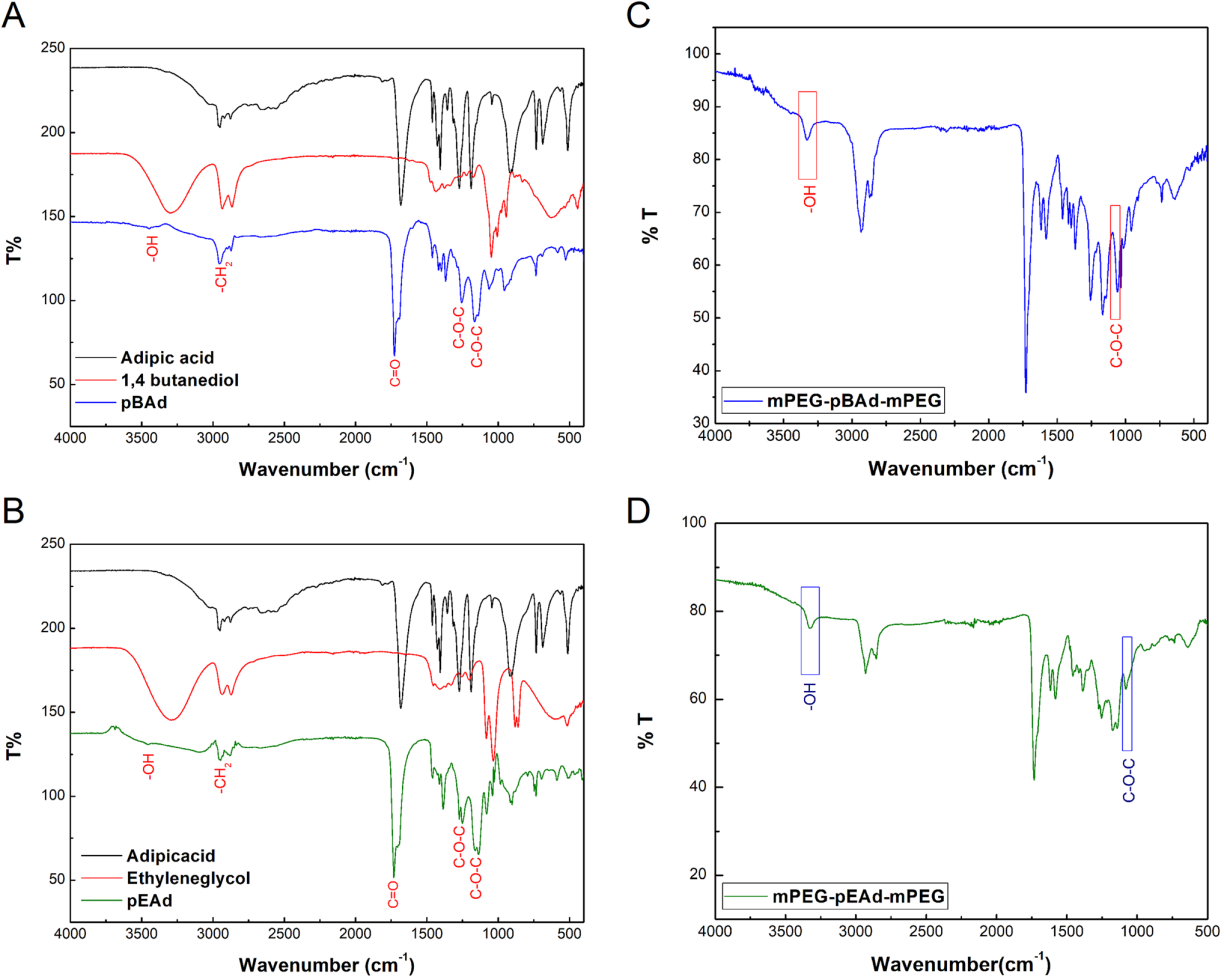
The FT-IR spectra of pBAd and pEAd hydrophobic polymers and the mPEG-modified amphiphilic triblock copolymer. The peaks of adipic acid, 1,4 butanediol, and pBAd (**A**), adipic acid, ethylene glycol, and pEAd (**B**). The FTIR peaks with mPEG-pBAd-mPEG amphiphilic triblock copolymer (**C**) and mPEG-pEAd-mPEG amphiphilic triblock copolymer (**D**)

The FT-IR spectra of pEAd and pBAd, synthesized from adipic acid, ethylene glycol, and 1,4-butanediol, were recorded in the wavenumber range of 400–4000 cm⁻^1^ to confirm the formation of the polyester and the end-group functionalities. In both spectra (Fig. 3 A and B), the strong absorption band observed at approximately 1730–1740 cm^−1^ is attributed to the ester carbonyl (C = O) stretching vibration, confirming successful esterification. The characteristic C–O–C stretching vibrations of the ester linkages appeared in the region of 1160–1250 cm^−1^, while the aliphatic –CH_2_– stretching vibrations were detected at around 2850–2950 cm^−1^. A broad absorption band centered at approximately 3400 cm^−1^ was assigned to the hydroxyl (–OH) stretching vibrations, indicating the presence of hydroxyl-terminated pEAd and pBAd chains (Nanaki et al. 2020). Following chain extension and functionalization with methoxy-poly(ethylene glycol) (mPEG) to obtain triblock amphiphilic copolymers, noticeable changes were observed in Fig. 3 C and D. The intensity of the –OH stretching band decreased markedly, suggesting the consumption of terminal hydroxyl groups during coupling with mPEG. Concurrently, the characteristic ether stretching vibration of PEG segments appeared as an intensified band around 1100 cm^−1^, confirming the successful incorporation of the hydrophilic mPEG block. The coexistence of ester carbonyl bands originating from the adipate segments and ether vibrations associated with mPEG clearly demonstrates the formation of triblock amphiphilic copolymers comprising hydrophobic polyester cores and hydrophilic PEG chains (Fan et al. 2021).

**Fig. 4:**
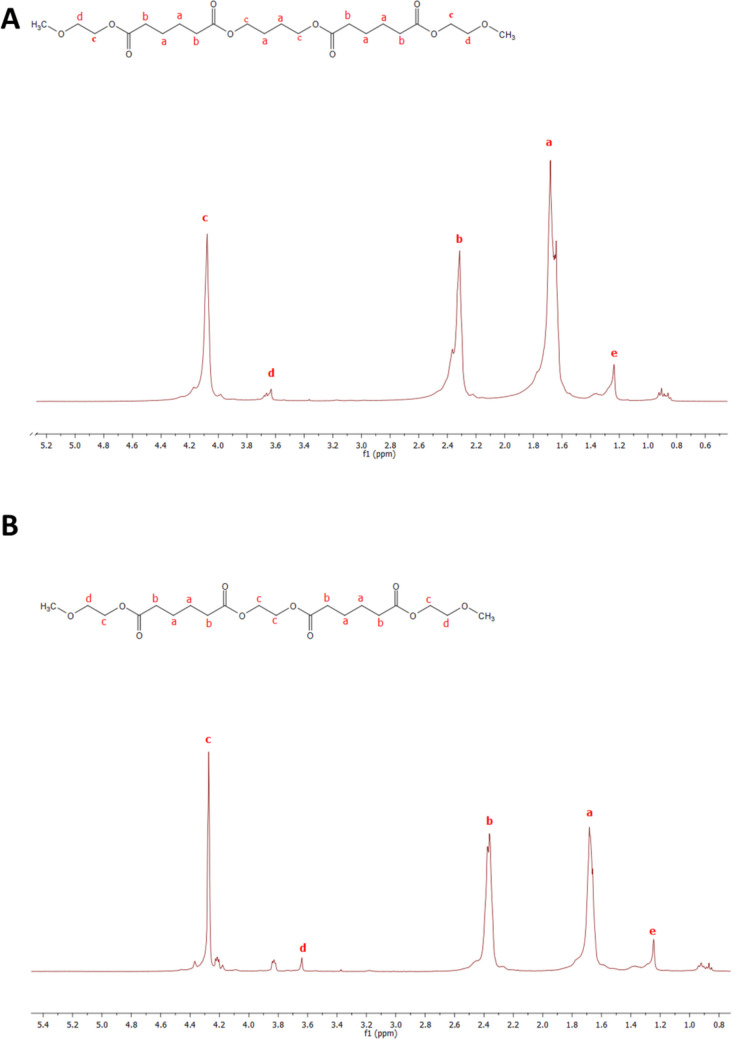
^1^H NMR spectra (400 MHz, CDCl_3_) of the synthesized amphiphilic triblock copolymers: **A** mPEG-pBAd-mPEG and **B** mPEG-pEAd-mPEG

The structural characterization of the polyadipate-based amphiphilic triblock copolymers (mPEG-pBAd-mPEG and mPEG-pEAd-mPEG) was performed by ^1^H NMR spectroscopy, and the corresponding spectra are presented in Fig. 4 A and B. The ^1^H NMR data provide a clear, structure-sensitive distinction between the two polyadipate-based triblock copolymers and directly support the design rationale based on diol chain length and flexibility. Notably, the diol-associated –O–CH_2_– signal (c) shifts from ~ 4.08 ppm in mPEG-pBAd-mPEG to ~ 4.27–4.28 ppm in mPEG-pEAd-mPEG, which is consistent with replacing the longer, more flexible butanediol segment with the shorter ethylene glycol segment (Debuissy et al. 2017). In contrast, the adipate carbonyl-adjacent methylene signal (b, ~ 2.36–2.34 ppm) and the internal aliphatic methylene envelope (a, ~ 1.68–1.66 ppm) remain in their expected ranges for aliphatic polyesters, indicating that the core polyadipate framework is preserved in both materials. Importantly, the observation of the PEG resonance at ~ 3.63 ppm (d) in both spectra supports the successful PEGylation of the polyadipate backbone, which is essential for the intended amphiphilic architecture and core–shell self-assembly behavior (Alexandridis and Lindman 2000).

From a functional perspective, these NMR-confirmed architectural differences are highly relevant to nanocarrier performance: the shorter ethylene glycol-based polyadipate segment is expected to yield a relatively more polar/compact hydrophobic domain, whereas the butanediol-based segment may provide increased chain flexibility within the hydrophobic core. Such distinctions can influence polymer–drug interactions, micellar/nanoparticle stability, and ultimately drug encapsulation and release behavior. Therefore, the ^1^H NMR results not only verify the success of the synthesis but also substantiate the comparative framework used throughout the study to relate polymer structure to physicochemical and biological performance.

**Fig. 5:**
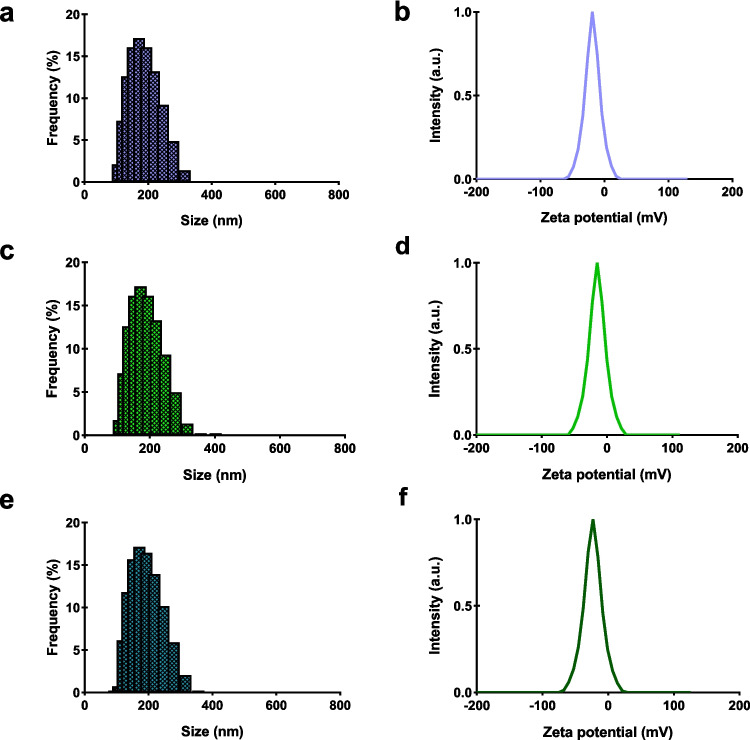
The particle size distribution graphs and zeta potential curves of polyadipate-based nanocarriers. Particle size distribution of BANPs (**a**), DBANPs (**c**), and IBANPs (**e**); zeta potential of BANPs (**b**), DBANPs (**d**), and IBANPs (**f**)

**Fig. 6:**
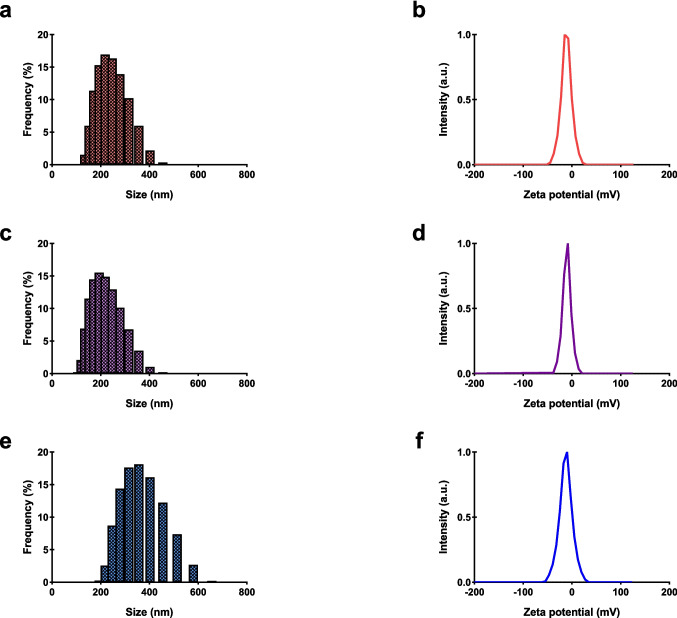
The particle size distribution graphs and zeta potential curves of polyadipate-based nanocarriers. Particle size distribution of EANPs (**a**), DEANPs (**c**), and IEANPs (**e**); zeta potential of EANPs (**b**), DEANPs (**d**), and IEANPs (**f**)

The production of polyadipate-based nanoparticles was performed using the double emulsion solvent evaporation method. Following the production of polyadipate-based nanoparticles, their physical properties, such as size distribution, zeta potential, and PDI, were characterized using DLS. The size distribution and zeta potential data of polyadipate-based nanoparticles are presented in Figs. 5 and 6 and are summarized in Table 1. Particle size is one of the key factors affecting the biological process of particle adhesion to the cell membrane, which includes particle adhesion to the cell membrane and cellular uptake. Nanoparticles larger than 200 nm can be phagocytosed by cells, while particles in the range of 100 to 200 nm are considered optimal for cellular uptake, and the lowest nanoparticle size limit for cellular uptake has been reported to be 50 nm (Banik et al. 2016; Behzadi et al. 2017). In this study, the particle sizes of non-drug-loaded polyadipate-based nanoparticles were revealed to be below 200 nm, with BANPs measuring 164.9 nm and EANPs measuring 197.6 nm. Upon loading with DOX and ICG, both BANP and EANP systems exhibited variations in particle size; however, all drug-loaded formulations maintained particle sizes under 250 nm, which is the preferred size for effective cellular uptake, systemic circulation, and drug delivery applications. These results demonstrated that drug-free and drug-loaded polyadipate-based nanoparticles synthesized may be suitable candidates for biomedical use, particularly in tumor targeting based on their particle size distributions.

**Table 1:** Particle size, PDI, and zeta potential values of BANPs, EANPs, DBANPs, DEANPs, IBANPs, and IEANPs

Sample	Size (nm)	PDI	Zeta potential (mV)
BANPs	164.9	0.170	− 20.2
DBANPs	162.6	0.109	− 18.5
IBANPs	158.2	0.114	− 20.2
EANPs	197.6	0.310	− 11.3
DEANPs	179.3	0.381	− 10.5
IEANPs	234.6	0.554	− 12.4

Tsachouridis et al. synthesized copolymers composed of poly(lactic acid), poly(lactic-co-glycolic acid), and poly(ethylene adipate) and subsequently prepared nanoparticles using the emulsion/solvent evaporation technique. The resulting nanoparticles had sizes ranging from 200 to 400 nm (Tsachouridis et al. 2022). Compared with the current study, although nanoparticles were produced using a similar polymeric system and technique, the nanoparticles produced here are smaller and may be more suitable for cellular uptake.

Moreover, the PDI is a key parameter that indicates the uniformity of the particle size distribution. A PDI value below 0.7 is generally accepted to represent a monodisperse system. The PDI values were obtained as 0.170 for BANPs and 0.310 for EANPs. The drug-loaded formulations also exhibited PDI values below 0.7, as summarized in Figs. 5 and 6, further validating the uniformity of the nanoparticle populations.

The zeta potential value, which is evidence of the colloidal stability of the nanoparticles and is an indicator of the surface charge of the nanoparticles, was also measured for all polyadipate-based nanoparticles produced as part of this research. The results show that they exhibited good colloidal stability (Kaplan et al. 2021; Khorrami et al. 2018).

Zeta potential measurements, which reflect the colloidal stability of nanoparticles, were also conducted on all polyadipate-based formulations. The results indicate that the nanoparticles exhibit stable colloidal characteristics, supporting their potential for biomedical applications. This indicates that all polyadipate-based nanoparticles exhibited stable dispersion in physiological media and effectively prevented nanoparticle aggregation, thereby enhancing bioavailability and therapeutic efficacy.

Compared to our previous study (Gökşen Tosun et al. 2023), the current data indicate that BANPs exhibit smaller particle sizes than PANPs (169.2 nm), whereas EANPs possess relatively larger sizes. Despite these differences in particle size, the polydispersity index (PDI), which reflects the monodispersity of the nanoparticle populations, and the zeta potential values, which indicate colloidal stability, remained comparable across all formulations. The observed differences in particle sizes among PANPs, BANPs, and EANPs can be attributed, in part, to the variations in the molecular weights and chemical structures of the hydrophobic polymers used during nanoparticle synthesis. Specifically, PANPs were synthesized using pPAd, a polymer with a molecular weight of 683 g/mol, while BANPs and EANPs were formulated using pBAd (952 g/mol) and pEAd (870 g/mol), respectively. The increasing molecular weight from pPAd to pEAd likely contributed to the enlargement of nanoparticle size due to enhanced chain entanglement and increased hydrophobic interactions during self-assembly. Additionally, the use of different diols—such as propanediol, butanediol, and ethylene glycol—may have influenced polymer chain flexibility, intermolecular spacing, and packing behavior, thereby affecting the final particle morphology and size distribution.

Encapsulation efficiency is a parameter defined as the amount of an active compound successfully encapsulated by nanocarriers. Achieving encapsulation at therapeutic dose levels is essential to ensure the effective delivery of the active agent to the intended target site. However, exceeding the therapeutic dose may result in adverse effects on healthy cells and tissues, potentially leading to pharmacokinetic imbalances. In the present study, the encapsulation efficiencies of polyadipate-based nanocarriers (BANPs and EANPs) were quantified using a spectrofluorometer, taking advantage of the intrinsic fluorescence of DOX·HCl and ICG, by measuring the fluorescence intensity of unencapsulated DOX in the supernatant after centrifugation. The results, presented in Table 2, show that BANPs achieved higher encapsulation efficiencies for both active agents compared to EANPs. Nevertheless, both formulations exhibited encapsulation efficiencies of more than 40%.

**Table 2:** Encapsulation efficiency (EE %) and drug loading (DL %) of polyadipate-based nanocarriers

Nanocarriers	EE (%)	DL (%)
DBANPs	58.0	0.58
IBANPs	46.0	0.46
DEANPs	44.5	0.45
IEANPs	41.2	0.42

The drug loading (DL%) values of the DOX-loaded nanocarriers were calculated using the equation described in the “Materials and methods” section, and the results are presented in Table 2. The DL% was determined to be 0.58% for DBANPs and 0.45% for DEANPs, indicating a slightly higher drug incorporation efficiency for DEANPs.

In polymeric micelle-based drug delivery systems, drug loading is largely determined by the polarity gradient established between the hydrophilic corona and the hydrophobic core. In amphiphilic triblock copolymer micelles, hydrophobic drugs preferentially reside within the lipophilic core, while molecules with intermediate polarity may be partially localized at the core–shell interface. Accordingly, determining drug loading capacity cannot be attributed solely to optimizing the preparation method; this is also closely related to the physicochemical compatibility between the drug and the polymeric core (Ahmad et al. 2014; Owen et al. 2012). In the present study, the relatively low DL% values obtained for DOX-loaded polyadipate-based nanocarriers can be attributed to the amphiphilic nature of DOX and the PEG-enhanced architecture of the triblock copolymers, which supports the formation of a highly hydrated shell. However, a review of the literature reveals that while drug loading in PEG-based micelle systems is generally limited, controlled release and an enhanced cellular uptake pathway are commonly reported to be sufficient to ensure therapeutic efficacy (Mai and Eisenberg 2012; Discher and Eisenberg 2002; Yuan et al. 2018). Here, polyadipate-based nanocarriers exhibited significant antiproliferative activity despite having a moderate drug loading percentage, demonstrating that drug loading efficiency alone does not determine biological performance. Instead, polymer-drug interactions, hydrophobic chain length, and micelle stability play decisive roles in determining overall therapeutic outcomes.

A comparison with our previous findings on the encapsulation efficiency of DOX HCl in PANPs (Gökşen Tosun et al. 2023) revealed that the BANPs drug delivery system developed in this study achieved a higher percentage of drug encapsulation.

Yuan et al. reported an encapsulation efficiency of approximately 90% for DOX·HCl when loading DOX into nanocarriers synthesized via nanoprecipitation using an mPEG-PLGA-PGlu copolymer (Yuan et al. 2018). In their system, the presence of functional moieties such as poly(L-glutamic acid) [P(Glu)] likely facilitated stronger electrostatic and hydrogen bonding interactions with DOX·HCl, contributing to the high encapsulation efficiency.

In contrast, the current study successfully encapsulated DOX·HCl into polyadipate-based nanocarriers (BANPs) without the incorporation of functional groups such as P(Glu). Although the encapsulation efficiency reported here is lower than that of Yuan et al., it demonstrates that effective drug loading can still be accomplished through hydrophobic interactions alone, particularly when using polymers with higher molecular weight and appropriate physicochemical characteristics.

**Fig. 7:**
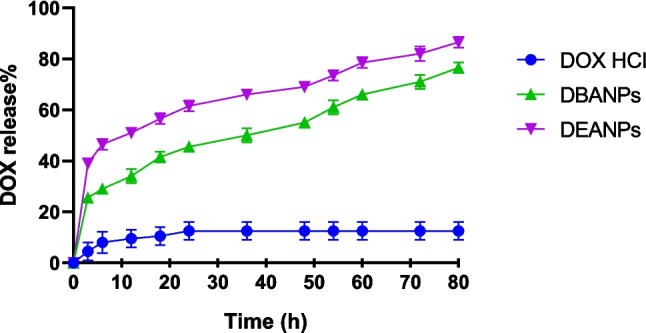
In vitro release profiles of DOX from polyadipate-based DOX-loaded nanocarriers compared to free DOX over 80 h in a simulated physiological environment (pH 7.4). Free DOX is shown as a reference representing immediate drug availability, while nanocarrier formulations demonstrate controlled and time-dependent release behavior

The primary goal of drug encapsulation is to minimize toxicity and adverse effects on healthy tissues prior to reaching the target site. Equally important is the drug release profile, which plays a critical role in ensuring therapeutic efficacy (Jadhav et al. 2024). The in vitro drug release profiles of the nanocarriers developed in this study are shown in Fig. 7. Analysis of the release curves reveals that both polyadipate-based nanocarriers exhibit distinct release behavior compared to free DOX. Free DOX was included in the release study as a reference control to represent immediate drug availability in the absence of a carrier system. Unlike encapsulated DOX, free DOX does not exhibit time-dependent release behavior, and its concentration remains essentially unchanged over the incubation period. Therefore, the comparison with free DOX was performed to highlight the controlled and sustained release characteristics imparted by the polyadipate-based nanocarriers rather than to imply a release mechanism for free DOX. Specifically, the drug release profiles of DBANPs and DEANPs followed a three-phase kinetic pattern: an initial burst release, a sustained release at a relatively constant rate, and a final progressive release phase. It was observed that DEANPs exhibited a higher overall release behavior throughout 80 h compared to DBANPs.

Notably, in our previous study (Gökşen Tosun et al. 2023), DPANPs demonstrated only 60% DOX release within 80 h. In contrast, the newly developed DBANPs and DEANPs achieved approximately 80% cumulative release within the first 48 h, highlighting their improved performance in terms of release efficiency and potential therapeutic impact.

**Fig. 8:**
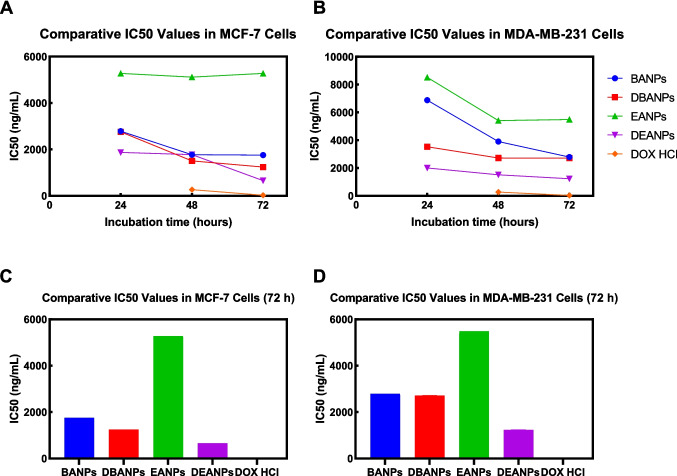
Time-dependent IC50 values of polyadipate-based nanocarriers in MCF-7 (**A**) and MDA-MB-231 (**B**) breast cancer cell lines after 24-, 48-, and 72-h incubation. Comparative IC_50_ values (ng/mL) of polyadipate-based nanocarriers in MCF-7 (**C**) and MDA-MB-231 (**D**) breast cancer cells after 72-h incubation. The figure highlights the differences in antiproliferative efficacy between drug-free (BANPs, EANPs) and DOX-loaded (DBANPs, DEANPs) formulations

The antiproliferative effects of the nano-formulations produced in this study in MCF-7 and MDA-MB-231 breast cancer cell lines were evaluated using the MTT assay. The half-maximum inhibitory concentration (IC50) values, calculated using GraphPad 8.1 software, are presented in Fig. 8.

Figure 8 summarizes the IC_50_ values of non-loaded polyadipate nanocarriers (BANPs and EANPs), and DOX-loaded polyadipate-based nanocarriers (DBANPs and DEANPs) in the MCF-7 and MDA-MB-231 breast cancer cell lines. As shown in Fig. 8 A and B, DBANPs and DEANPs showed a significant time-dependent decrease in IC_50_ values in both cell lines, while BANPs and EANPs maintained high IC_50_ values with minimal variation over time, exhibiting negligible intrinsic cytotoxicity. These results can be attributed to the biocompatibility of polyadipate-based nanocarriers. Among DOX-loaded nanocarriers, DEANPs consistently showed lower IC_50_ values than DBANPs.

Comparative analysis at 72 h (Fig. 8 C and D) in both MCF-7 and MDA-MB-231 cells showed that the DBANP and DEANP nanocarriers demonstrated significantly enhanced antiproliferative effects compared to BANPs, EANPs, and free DOX. Although MDA-MB-231 cells exhibited generally higher IC_50_ values, consistent with their more aggressive phenotype, the relative performance of the formulations remained unchanged.

**Fig. 9:**
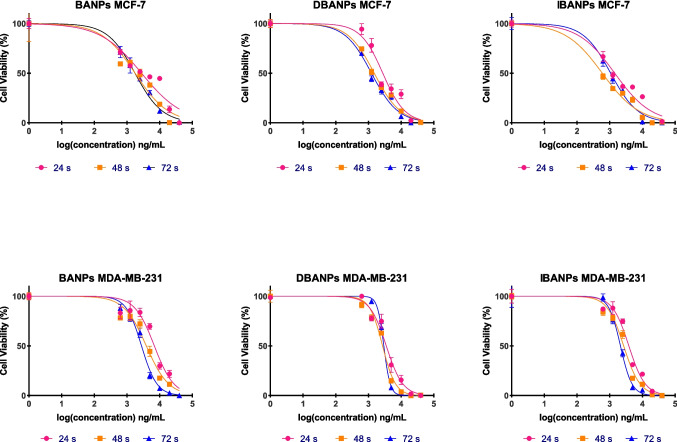
Comparative antiproliferative effects of mPEG-functionalized poly(butylene adipate) triblock copolymer–based nanocarriers (BANP, DBANP, and IBANP) on MCF-7 and MDA-MB-231 breast cancer cell lines following 24-h, 48-h, and 72-h incubation

**Fig. 10:**
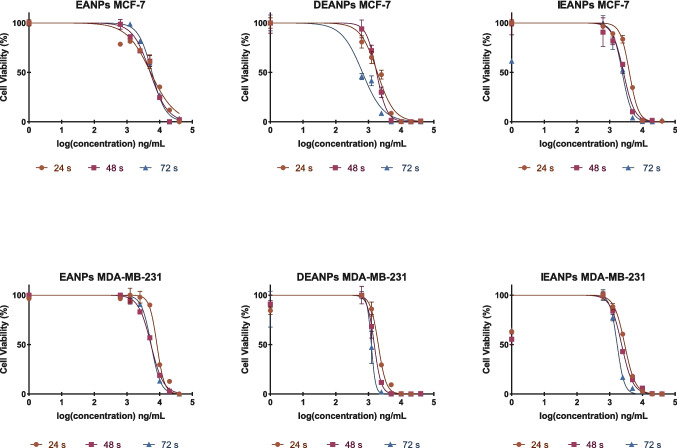
Comparative antiproliferative effects of mPEG-functionalized poly(ethylene adipate) triblock copolymer–based nanocarriers (EANPs, DEANPs, and IEANPs) on MCF-7 and MDA-MB-231 breast cancer cell lines following 24-h, 48-h, and 72-h incubation

The cytotoxic and antiproliferative effects of non-loaded BANPs and EANPs, DOX-loaded DBANPs, DEANPs, and ICG-loaded DEANPs, and ICG-loaded IBANPs, and IEANPs were evaluated through MTT assay, employing seven-point serial dilutions starting at 40 µg/mL. Logarithmic graphs of % cell viability versus concentration at three different time points—24 h, 48 h, and 72 h—presented in Figs. 9 and 10 were plotted using GraphPad 8.1 software, providing insight into the potential cytotoxicity of the nanocarrier matrices themselves.

These results demonstrate that empty polyadipate-based nanocarriers (BANPs and EANPs) exhibit low toxicity after 72 h of incubation in both cell lines. This finding is consistent with the biocompatible properties of aliphatic polyester-based structures, and the results reported in the literature (Khorrami et al. 2018; Ahmad et al. 2014), which support the biocompatibility of the synthesized systems.

A significant concentration-dependent decrease in cell viability was observed in DOX-loaded systems. After 72 h of incubation, the calculated IC_50_ values for the MCF-7 cell line were found to be BANPs 1759 ng/mL, DBANPs 1243 ng/mL, EANPs 5277 ng/mL, and DEANPs 656.4 ng/mL. Similarly, in the MDA-MB-231 cell line, the concentrations of BANPs, DBANPs, EANPs, and DEANPs were 2788 ng/mL, 2710 ng/mL, 5488 ng/mL, and 1230 ng/mL, respectively. These results demonstrated that DOX-loaded nanocarriers produce a more controlled and effective cytotoxic effect compared to free DOX. It was also observed that EANPs exhibit higher biocompatibility than BANPs, whereas their DOX-loaded forms (DEANPs) caused a significant decrease in cell viability. The time-dependent drug release profile of DEANPs showed a higher initial burst effect compared to DBANPs. This rapid initial release explains the difference in cytotoxic effects observed between EANPs and DEANPs. Based on the results, EANPs may have facilitated faster drug diffusion in the initial phase due to their more hydrophilic nature. Consequently, the lower IC_50_ value at 72 h compared to DBANPs may also be attributed to the effects of polymer chain mobility and surface morphology on release (Ma et al. 2016). Furthermore, the MDA-MB-231 cell line was observed to be more resistant to DOX-loaded polyadipate-based nanocarriers than the MCF-7 cell line. This may be due to the different endocytosis capacities and drug resistance mechanisms (e.g., P-gp expression) of triple-negative breast cancer cells (Famta et al. 2021).

**Fig. 11:**
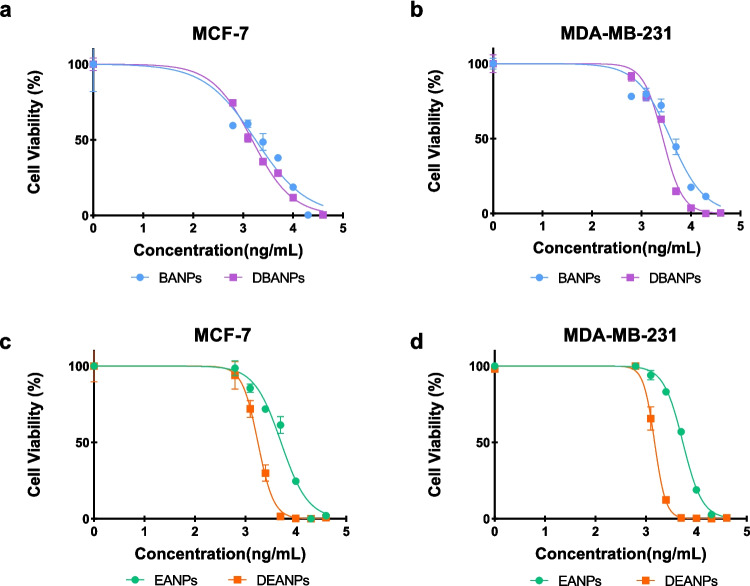
The cytotoxic logarithmic curves of polyadipate-based nanocarriers in breast cancer cell lines over a 48-h period: cytotoxic effect of BANPs and DBANPs on MCF-7 (**a**), cytotoxic effect of BANPs and DBANPs on MDA-MB-231 (**b**), cytotoxic effect of EANPs and DEANPs on MCF-7 (**c**), cytotoxic effect of EANPs and DEANPs on MDA-MB-231 (**d**)

Cell viability was comparatively evaluated after treatment with different concentrations of empty and drug-loaded nanoparticles. The cytotoxic effects of both non-drug-loaded (BANPs and EANPs) and DOX-loaded (DBANPs and DEANPs) polyadipate-based nanocarriers after 48 h of incubation were comparatively evaluated and are presented in Fig. 11. As expected, DOX-loaded nano-formulations exhibited significantly higher cytotoxicity compared to their unloaded nanocarriers. Notably, the difference between EANPs and DEANPs was particularly pronounced, which aligns well with the observed drug release kinetics.

These findings confirm that DOX was effectively encapsulated within the nanocarriers and successfully released over time, thereby enhancing its antiproliferative effect. Furthermore, the results suggest a potential clinical advantage: encapsulating DOX within polyadipate-based nanocarriers may reduce its systemic toxicity by allowing for controlled and localized drug release, thereby minimizing damage to healthy tissues while maintaining therapeutic efficacy.

**Fig. 12:**
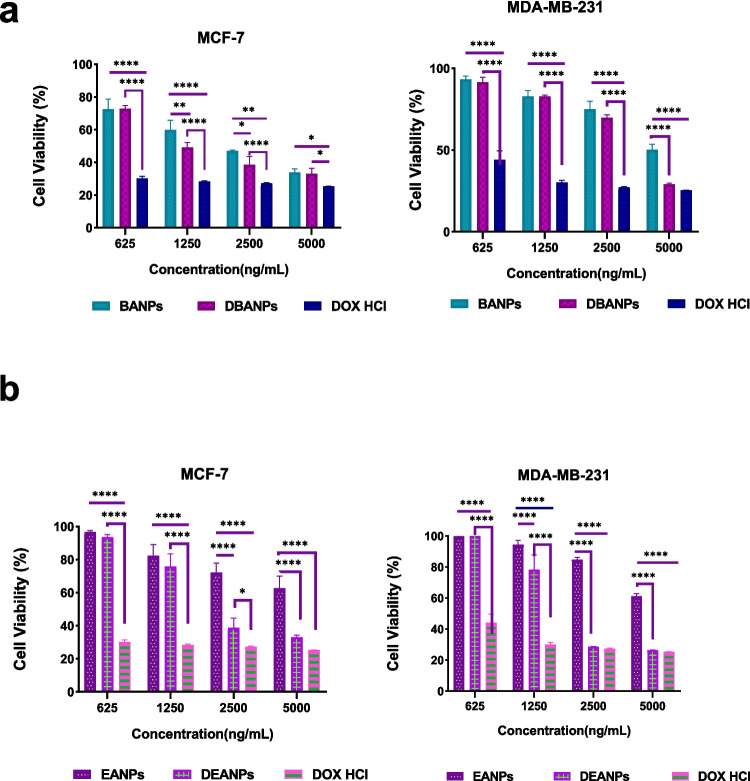
Comparison of cell viability percentages after 48-h incubation with free DOX and DOX-loaded nanoparticles (DBANPs (**a**), DEANPs (**b**)) at various concentrations. A statistically significant difference was observed between treatment groups (*p* < 0.005)

The comparative antiproliferative effects of empty polyadipate-based nanocarriers (BANPs and EANPs), doxorubicin-loaded polyadipate nanocarriers (DBANPs and DEANPs), and free doxorubicin hydrochloride (DOX HCl) against MCF-7 and MDA-MB-231 breast cancer cell lines after 48 h of incubation at four different concentrations (625–5000 ng/mL) are shown in Fig. 12. A dose-dependent decrease in cell viability was observed for all formulations in both cell lines. This is consistent with the typical cytotoxic profile of DOX and its nano-formulations (Ghezzi et al. 2021; Liu et al. 2024). Empty polyadipate-based nanoparticles maintained cell viability even at the highest concentration, exhibiting negligible toxicity. The finding of cell viability above 50% even at the highest concentration, where EANPs exhibited lower cytotoxicity than BANPs, demonstrates the biocompatibility and non-toxicity of these polyadipate-based nanocarriers. This is consistent with previous findings on aliphatic polyester-based biodegradable systems (Kreua-ongarjnukool et al. 2022). DBANPs and DEANPs, on the other hand, exhibited significantly increased cytotoxicity compared to their empty nanocarriers at all tested concentrations (*p* < 0.001). This increased inhibitory effect on cells can be attributed to the sustained intracellular release of DOX from the polyadipate-based nanocarriers’ matrix. The time-dependent release profile of DEANPs differed from that of DBANPs, exhibiting a more pronounced initial burst effect. This rapid release at the early stage may account for the greater difference observed in the cytotoxic responses of EANPs and DEANPs toward MCF-7 and MDA-MB-231 cell lines compared to the difference between BANPs and DBANPs. At the same time, free DOX HCl exhibited the greatest immediate cytotoxic effect, rapidly reducing cell viability to less than 30% at concentrations of 1250 ng/mL or higher. However, the sudden decrease in viability is often associated with immediate intracellular uptake and acute toxicity, which may limit therapeutic efficacy and increase systemic side effects in clinical applications (Bhadran et al. 2025). In contrast, the DBANP and DEANP groups demonstrated a more controlled yet comparably effective cytotoxic profile, indicating that DOX nanoencapsulation reduces immediate cytotoxicity while maintaining therapeutic efficacy and potentially prevents the side effects of DOX on healthy cells and tissues.

When comparing the cytotoxic effects of DOX-loaded polyadipate-based nanocarriers in breast cancer cells on a cell-by-cell basis, the more aggressive phenotype of MDA-MB-231 triple-negative breast cancer cells may have led to higher DOX sensitivity (Han et al. 2019) and to lower cell viability compared to MCF-7 cells. This difference may be attributed to the higher metabolic activity and enhanced membrane permeability of the MDA-MB-231 cell phenotype, which favors cellular uptake of nanoparticles. The findings confirm that polyadipate-based nanocarriers are effective and biocompatible drug delivery systems for DOX that may maintain cytotoxic activity against breast cancer cells while minimizing undesirable toxicity.

**Fig. 13:**
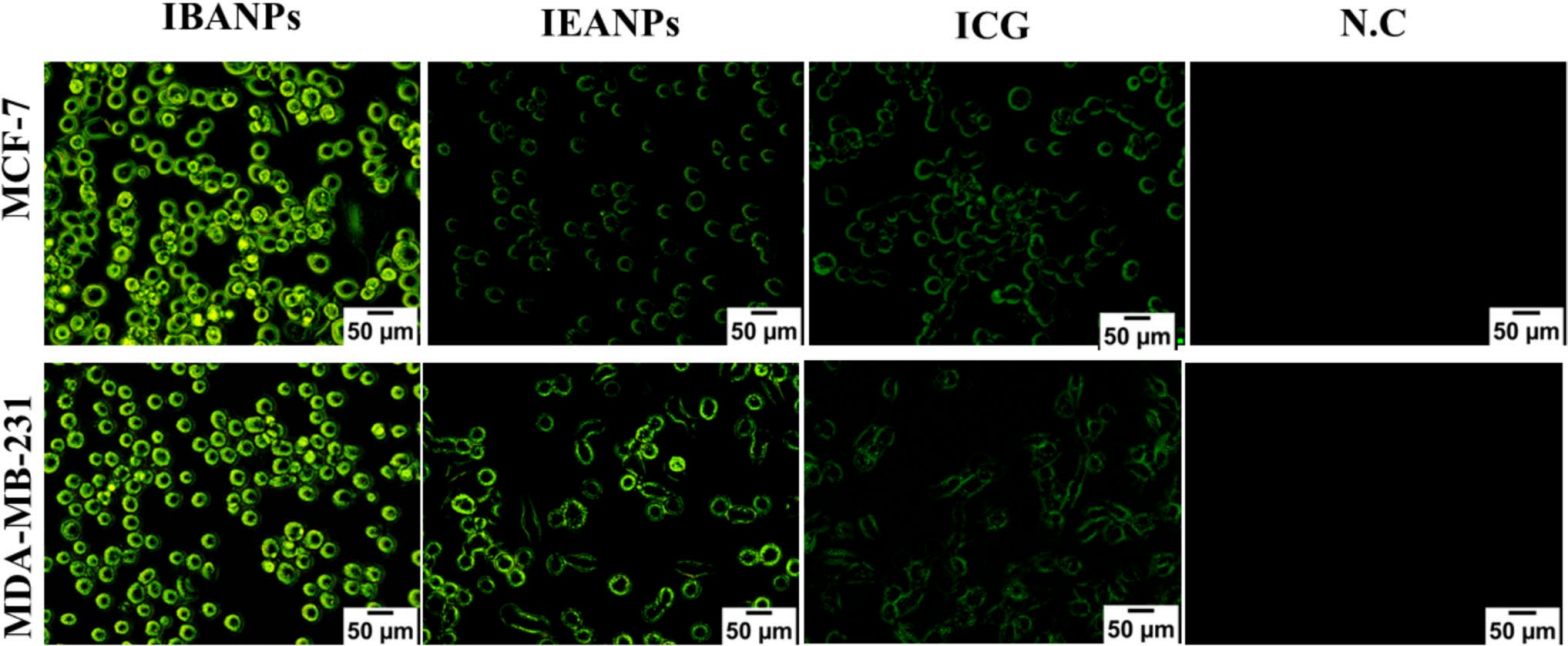
Visualization of intracellular accumulation of IBANPs and IEANPs versus free ICG in MCF-7 and MDA-MB-231 cells using fluorescence microscopy to assess cellular uptake efficiency

To evaluate the cellular uptake of polyadipate-based nanocarriers, the nanocarriers were loaded with indocyanine green (ICG), which served as both a model drug and a fluorescent probe for visualization. ICG-loaded polyadipate-based nanocarriers were administered to MCF-7 and MDA-MB-231 cell lines, and cellular uptake was assessed by fluorescence microscopy. Figure 13 presents representative fluorescence microscopy images comparing ICG-loaded polyadipate-based nanocarriers with free ICG and a negative control group (untreated cells). As shown in the images, the observed fluorescence signals are attributable to ICG, as no fluorescence was detected in the negative control group.

The cellular uptake study clearly demonstrated that polyadipate-based nanocarriers efficiently facilitate the internalization of ICG in both MCF-7 and MDA-MB-231 cancer cell lines, outperforming free ICG. This enhancement can be attributed to the nanoparticulate structure, which increases membrane interaction and endocytosis internalization, consistent with previous reports indicating that nanocarrier-mediated delivery systems improve drug uptake compared to free small molecules (Ma et al. 2016; Zhao et al. 2020). The absence of fluorescence in the untreated control group confirms that the observed signals originate solely from ICG. A comparative evaluation between IBANPs and IEANPs indicated that IBANPs penetrated more effectively into the cytoplasmic compartments, suggesting a higher affinity for cellular membranes, potentially due to differences in their surface chemistry and hydrophobic interactions. Interestingly, although IEANPs exhibited relatively lower penetration compared to IBANPs, their uptake was still significantly greater than that of free ICG. This finding aligns with studies showing that even moderate modifications to nanoparticle composition can substantially enhance intracellular delivery efficiency (Xu et al. 2015). In particular, the observed enhanced uptake of IEANPs in MDA-MB-231 cells compared to MCF-7 cells may be related to the higher metabolic activity and endocytosis capacity of triple-negative breast cancer cells, which have been reported to internalize nanoparticles more efficiently compared to hormone receptor–positive cells (Xu et al. 2015). Fluorescence microscopy images qualitatively confirmed enhanced intracellular localization of ICG when delivered via polyadipate-based nanocarriers compared to free ICG. These observations support the improved cellular interaction of the nanocarriers and complement the antiproliferative activity data, although quantitative uptake analysis was beyond the scope of the present study. These results strongly support the potential of polyadipate-based nanocarriers for intracellular delivery of poorly bioavailable drugs, such as ICG or chemotherapeutics, thereby offering a promising platform for targeted cancer therapy.

## Conclusion

In this study, amphiphilic triblock copolymers of mPEG-polyadipate-mPEG were produced using two glycols (ethylene glycol and butane diol) as novel nanocarrier materials for the controlled delivery of potential drug substances (DOX and ICG) used for cancer therapy. These materials were characterized in terms of their chemical structures and biological effects:

- The produced materials were under investigation for the functional groups in the backbones. FT-IR analyses confirmed the presence of ester bonds in polyadipates and –NH stretching vibrations in bonding groups, demonstrating a successful copolymerization process.
- Polyadipate-based nanocarriers exhibited colloidal stability and formed uniform and monodisperse structures with a narrow particle distribution.
- DOX release experiments conducted under physiological conditions (pH 7.4) revealed that both systems exhibited a pronounced initial burst effect, followed by a controlled and prolonged drug release behavior.

Cell culture analyses demonstrated that the DEANPs system, in particular, had significant antiproliferative effects in MCF-7 and MDA-MB-231 breast cancer cell lines, while the BANPs and EANPs nanocarriers exhibited high biocompatibility. Furthermore, a lower but more effective toxicity profile compared to the free form of DOX suggests that these systems may reduce side effects in healthy cells and tissues.

In conclusion, the two polyadipate-based nanocarrier systems developed in this study—BANPs and EANPs—yielded promising results in both structural and biological evaluations. These systems are strong candidates as targeted drug delivery platforms for future cancer treatment due to their high biocompatibility, controlled release properties, and selective cytotoxicity.

## Data Availability

The datasets generated and/or analyzed during the current study are available from the corresponding authors on reasonable request.

## References

[CR1] Ahmad Z, Shah A, Siddiq M, Kraatz H-B (2014) Polymeric micelles as drug delivery vehicles. RSC Adv 4:17028–17038. 10.1039/C3RA47370H

[CR2] Alexandridis P, Lindman B (2000) Amphiphilic block copolymers: self-assembly and applications (studies in surface science and catalysis). Elsevier

[CR3] Alkan C, Gökşen Tosun N, Kaplan Ö (2023) Synthesis and characterization of dicarboxylic acid esters of 1-hexadecanol for a thermal energy storage application range of 50–55 °C. Energy Technol. 10.1002/ente.202300104

[CR4] Axioti E, Dixon EG, Jepras T et al (2025) Enzymatic synthesis of functional PEGylated adipate copolymers. ChemPlusChem 90:e202400668. 10.1002/cplu.202400668 40019286 10.1002/cplu.202400668 PMC12105458

[CR5] Banik BL, Fattahi P, Brown JL (2016) Polymeric nanoparticles: the future of nanomedicine. Wires Nanomed Nanobiotechnol 8:271–299. 10.1002/wnan.1364 10.1002/wnan.1364 26314803

[CR6] Beach MA, Nayanathara U, Gao Y et al (2024) Polymeric nanoparticles for drug delivery. Chem Rev 124:5505–5616. 10.1021/acs.chemrev.3c00705 38626459 10.1021/acs.chemrev.3c00705 PMC11086401

[CR7] Behzadi S, Serpooshan V, Tao W et al (2017) Cellular uptake of nanoparticles: journey inside the cell. Chem Soc Rev 46:4218–4244. 10.1039/C6CS00636A 28585944 10.1039/c6cs00636a PMC5593313

[CR8] Bhadran A, Polara H, Babanyinah GK et al (2025) Advances in doxorubicin chemotherapy: emerging polymeric nanocarriers for drug loading and delivery. Cancers (Basel). 10.3390/cancers17142303 40723187 10.3390/cancers17142303 PMC12293504

[CR9] Bu Y, Ma J, Bei J, Wang S (2019) Surface modification of aliphatic polyester to enhance biocompatibility. Front Bioeng Biotechnol 7:98. 10.3389/fbioe.2019.00098 31131273 10.3389/fbioe.2019.00098 PMC6509149

[CR10] Chaudhary AK, Beckman EJ, Russell AJ (1997) Biocatalytic polyester synthesis: analysis of the evolution of molecular weight and end group functionality. Biotechnol Bioeng 55:227–239. 10.1002/(SICI)1097-0290(19970705)55:1<227::AID-BIT23>3.0.CO;2-H 18636460 10.1002/(SICI)1097-0290(19970705)55:1<227::AID-BIT23>3.0.CO;2-H

[CR11] Chesterman J, Zhang Z, Ortiz O et al (2020) Biodegradable polymers. In: Lanza R, Langer R, Vacanti JP, Atala ABT-P of TE (Fifth E (eds) Principles of tissue engineering. Academic Press, pp 317–342. 10.1016/B978-0-12-818422-6.00019-8

[CR12] Chime SA, Momoh MA (2025) PEGylated nanocarriers for drug delivery applications BT - PEGylated nanocarriers in medicine and pharmacy. In: Jain NK (ed) Tekade RK. Springer Nature Singapore, Singapore, pp 107–136. 10.1007/978-981-97-7316-9_4

[CR13] Das A, KN C, Salins SS et al (2025) Poly (butylene adipate-co-terephthalate) (PBAT) in biomedical applications: a comprehensive review of material properties, fabrication methods, and biofunctional potential. Mater Res Express 12:62002. 10.1088/2053-1591/ade498

[CR14] Debuissy T, Sangwan P, Pollet E, Avérous L (2017) Study on the structure-properties relationship of biodegradable and biobased aliphatic copolyesters based on 1,3-propanediol, 1,4-butanediol, succinic and adipic acids. Polymer 122:105–116. 10.1016/j.polymer.2017.06.045

[CR15] Discher DE, Eisenberg A (2002) Polymer vesicles. Science (80- ) 297:967–973. 10.1126/science.1074972 10.1126/science.1074972 12169723

[CR16] Erden Tayhan S (2024) A study with cancer stem cells and three-dimensional tumoroids: investigation of the combined effects of 5-fluorouracil and doxorubicin in breast cancer. Med Oncol 41:185. 10.1007/s12032-024-02423-4 38910198 10.1007/s12032-024-02423-4 PMC11194218

[CR17] Famta P, Shah S, Chatterjee E et al (2021) Exploring new horizons in overcoming P-glycoprotein-mediated multidrug-resistant breast cancer via nanoscale drug delivery platforms. Curr Res Pharmacol Drug Discov 2:100054. 10.1016/j.crphar.2021.100054 34909680 10.1016/j.crphar.2021.100054 PMC8663938

[CR18] Fan X, Pu Z, Zhu M et al (2021) Solvent-free synthesis of PEG modified polyurethane solid-solid phase change materials with different Mw for thermal energy storage. Colloid Polym Sci 299:835–843. 10.1007/s00396-020-04804-3

[CR19] Ghezzi M, Pescina S, Padula C et al (2021) Polymeric micelles in drug delivery: an insight of the techniques for their characterization and assessment in biorelevant conditions. J Control Release 332:312–336. 10.1016/j.jconrel.2021.02.031 33652113 10.1016/j.jconrel.2021.02.031

[CR20] Gökşen Tosun N (2024) Enhancing therapeutic efficacy in breast cancer: a study on the combined cytotoxic effects of doxorubicin and MPC-3100. Naunyn Schmiedebergs Arch Pharmacol 397:3249–3259. 10.1007/s00210-023-02807-9 37917369 10.1007/s00210-023-02807-9

[CR21] Gökşen Tosun N, Kaplan Ö (2025) Dual targeting of HSP90 and BCL-2 in breast cancer cells using inhibitors BIIB021 and ABT-263. Breast Cancer Res Treat 210:493–506. 10.1007/s10549-024-07587-1 39779635 10.1007/s10549-024-07587-1 PMC11930872

[CR22] Gökşen Tosun N, Erden Tayhan S, Gökçe İ, Alkan C (2023) Doxorubicin-loaded mPEG-pPAd-mPEG triblock polymeric nanoparticles for drug delivery systems: preparation and *in vitro* evaluation. J Mol Struct 1291:135959. 10.1016/j.molstruc.2023.135959

[CR23] Guo C, Lin L, Wang Y et al (2025a) Nano drug delivery systems for advanced immune checkpoint blockade therapy. Theranostics 15:5440–5480. 10.7150/thno.112475 40303342 10.7150/thno.112475 PMC12036873

[CR24] Guo Z, Xiao Y, Wu W et al (2025b) Metal–organic framework-based smart stimuli-responsive drug delivery systems for cancer therapy: advances, challenges, and future perspectives. J Nanobiotechnology 23:157. 10.1186/s12951-025-03252-x 40022098 10.1186/s12951-025-03252-x PMC11871784

[CR25] Han J, Lim W, You D et al (2019) Chemoresistance in the human triple-negative breast cancer cell line MDA-MB-231 induced by doxorubicin gradient is associated with epigenetic alterations in histone deacetylase. J Oncol 2019:1345026. 10.1155/2019/1345026 31275376 10.1155/2019/1345026 PMC6582875

[CR26] Jadhav V, Roy A, Kaur K et al (2024) Recent advances in nanomaterial-based drug delivery systems. Nano-Struct Nano-Objects 37:101103. 10.1016/j.nanoso.2024.101103

[CR27] Kaplan Ö, Gökşen Tosun N, Özgür A et al (2021) Microwave-assisted green synthesis of silver nanoparticles using crude extracts of *Boletus edulis* and *Coriolus versicolor*: characterization, anticancer, antimicrobial and wound healing activities. J Drug Deliv Sci Technol 64:102641. 10.1016/j.jddst.2021.102641

[CR28] Kaplan Ö, Gökşen Tosun N, Gökçe İ, Alkan C (2023) Diacid esters of 1-dodecanol as new alternatives to solid-liquid phase change materials for solar heat storage systems. Energy Sources Part A Recover Util Environ Eff 45:608–622. 10.1080/15567036.2023.2172103

[CR29] Kaplan Ö, Gök MK, Pekmez M et al (2024) Development of recombinant protein-based nanoparticle systems for inducing tumor cell apoptosis: *in vitro* evaluation of their cytotoxic and apoptotic effects on cancer cells. J Drug Deliv Sci Technol 95:105565. 10.1016/j.jddst.2024.105565

[CR30] Kataoka K, Harada A, Nagasaki Y (2001) Block copolymer micelles for drug delivery: design, characterization and biological significance. Adv Drug Deliv Rev 47:113–131. 10.1016/S0169-409X(00)00124-1 11251249 10.1016/s0169-409x(00)00124-1

[CR31] Khorrami S, Zarrabi A, Khaleghi M et al (2018) Selective cytotoxicity of green synthesized silver nanoparticles against the MCF-7 tumor cell line and their enhanced antioxidant and antimicrobial properties. Int J Nanomedicine 13:8013–8024 30568442 10.2147/IJN.S189295 PMC6267361

[CR32] Kim J, Harper A, McCormack V et al (2025) Global patterns and trends in breast cancer incidence and mortality across 185 countries. Nat Med 31:1154–1162. 10.1038/s41591-025-03502-3 39994475 10.1038/s41591-025-03502-3

[CR33] Kreua-ongarjnukool N, Soomherun N, Thumsing Niyomthai S, Chumnanvej S (2022) Aliphatic polyester nanoparticles for drug delivery systems. In: Ahmad U, Haider MF, Akhtar J (eds) Smart Drug Delivery. IntechOpen, London

[CR34] Li D, Jin P, Cai Y et al (2025) Clinical significance of lipid pathway-targeted therapy in breast cancer. Front Pharmacol 15:1514811. 10.3389/fphar.2024.1514811 10.3389/fphar.2024.1514811 PMC11743736 39834807

[CR35] Liu Y, Craig DQM, Parhizkar M (2024) Controlled release of doxorubicin from Poly-(D,L-lactide-co-glycolide) (PLGA) nanoparticles prepared by coaxial electrospraying. Int J Pharm 666:124724. 10.1016/j.ijpharm.2024.124724 39312984 10.1016/j.ijpharm.2024.124724

[CR36] Long J, Shao T, Wang Y et al (2025) PEGylation of dipeptide linker improves therapeutic index and pharmacokinetics of antibody-drug conjugates. Bioconjug Chem 36:179–189. 10.1021/acs.bioconjchem.4c00392 39832173 10.1021/acs.bioconjchem.4c00392

[CR37] Ma Y, Huang J, Song S et al (2016) Cancer-targeted nanotheranostics: recent advances and perspectives. Small 12:4936–4954. 10.1002/smll.201600635 27150247 10.1002/smll.201600635

[CR38] Mai Y, Eisenberg A (2012) Self-assembly of block copolymers. Chem Soc Rev 41:5969–5985. 10.1039/C2CS35115C 22776960 10.1039/c2cs35115c

[CR39] Makharadze D, Del Valle LJ, Katsarava R, Puiggalí J (2025) The art of PEGylation: from simple polymer to sophisticated drug delivery system. Int J Mol Sci. 10.3390/ijms26073102 40243857 10.3390/ijms26073102 PMC11988339

[CR40] Mi J, Li Y, Wang W et al (2025) Synthesis and characterization of erythritol-modified poly(butylene adipate -co- terephthalate). Mater Today Commun 42:111318. 10.1016/j.mtcomm.2024.111318

[CR41] Musielak E, Krajka-Kuźniak V (2025) Lipidic and inorganic nanoparticles for targeted glioblastoma multiforme therapy: advances and strategies. Micro. 10.3390/micro5010002

[CR42] Nanaki S, Viziridou A, Zamboulis A, et al (2020) New biodegradable poly ( l -lactide ) -block- for long-acting injectables of naltrexone drug. Polymers (Basel) 12:852. 10.3390/polym12040852 10.3390/polym12040852 PMC7240759 32272700

[CR43] Owen SC, Chan DPY, Shoichet MS (2012) Polymeric micelle stability. Nano Today 7:53–65. 10.1016/j.nantod.2012.01.002

[CR44] Panda PK, Purohit A, Mishra S et al (2024) Inorganic nanoparticles-based strategies for cancer immunotherapy BT - nanotechnology based strategies for cancer immunotherapy: concepts, design, and clinical applications. In: Pandey V, Mishra N (eds) Sharma R. Springer Nature Singapore, Singapore, pp 327–353

[CR45] Sabzehali S (2025) Nanomaterials in drug delivery systems: challenges and perspectives. Eurasian J Chem Med Pet Res 4:96–110

[CR46] Saripilli R, Sharma DK (2025) Nanotechnology-based drug delivery system for the diagnosis and treatment of ovarian cancer. Discov Oncol 16:422. 10.1007/s12672-025-02062-9 40155504 10.1007/s12672-025-02062-9 PMC11953507

[CR47] Spriet J, Ben MA, Mailliez A et al (2025) Breast magnetic resonance imaging patterns of tumor regression after neoadjuvant chemotherapy and immunotherapy in early triple-negative breast cancer patients: prediction of pathological response and performance of ultrafast sequences. Breast Cancer Res Treat 212:195–203. 10.1007/s10549-025-07650-5 40434685 10.1007/s10549-025-07650-5

[CR48] Torchilin VP (2001) Structure and design of polymeric surfactant-based drug delivery systems. J Control Release 73:137–172. 10.1016/S0168-3659(01)00299-1 11516494 10.1016/s0168-3659(01)00299-1

[CR49] Tsachouridis K, Christodoulou E, Zamboulis A et al (2022) Evaluation of poly(lactic acid)/ and poly(lactic-co-glycolic acid)/ poly(ethylene adipate) copolymers for the preparation of paclitaxel loaded drug nanoparticles. J Drug Deliv Sci Technol 77:103918. 10.1016/j.jddst.2022.103918

[CR50] Ward RS, Jones RL (2011) 1.125 - polyurethanes and silicone polyurethane copolymers. In: Ducheyne PBT-CB (ed). Elsevier, Oxford, pp 431–477. 10.1016/B978-0-08-055294-1.00272-5

[CR51] Xiong X, Zheng L-W, Ding Y et al (2025) Breast cancer: pathogenesis and treatments. Signal Transduct Target Ther 10:49. 10.1038/s41392-024-02108-4 39966355 10.1038/s41392-024-02108-4 PMC11836418

[CR52] Xu X, Ho W, Zhang X et al (2015) Cancer nanomedicine: from targeted delivery to combination therapy. Trends Mol Med 21:223–232. 10.1016/j.molmed.2015.01.001 25656384 10.1016/j.molmed.2015.01.001 PMC4385479

[CR53] Yuan J-D, ZhuGe D-L, Tong M-Q et al (2018) pH-sensitive polymeric nanoparticles of mPEG-PLGA-PGlu with hybrid core for simultaneous encapsulation of curcumin and doxorubicin to kill the heterogeneous tumour cells in breast cancer. Artif Cells, Nanomedicine, Biotechnol 46:302–313. 10.1080/21691401.2017.1423495 10.1080/21691401.2017.1423495 29301415

[CR54] Zhao Z, Ukidve A, Kim J, Mitragotri S (2020) Targeting strategies for tissue-specific drug delivery. Cell 181:151–167. 10.1016/j.cell.2020.02.001 32243788 10.1016/j.cell.2020.02.001

